# Children of Few Words: Relations Among Selective Mutism, Behavioral Inhibition, and (Social) Anxiety Symptoms in 3- to 6-Year-Olds

**DOI:** 10.1007/s10578-015-0547-x

**Published:** 2015-04-05

**Authors:** Peter Muris, Eline Hendriks, Suili Bot

**Affiliations:** Clinical Psychological Science, Faculty of Psychology and Neuroscience, Maastricht University, P.O. Box 616, 6200 MD Maastricht, The Netherlands; Stellenbosch University, Stellenbosch, South Africa

**Keywords:** Selective mutism, Behavioral inhibition, (Social) anxiety, Children

## Abstract

Children with selective mutism (SM) fail to speak in specific public situations (e.g., school), despite speaking normally in other situations (e.g., at home). The current study explored the phenomenon of SM in a sample of 57 non-clinical children aged 3–6 years. Children performed two speech tasks to assess their absolute amount of spoken words, while their parents completed questionnaires for measuring children’s levels of SM, social anxiety and non-social anxiety symptoms as well as the temperament characteristic of behavioral inhibition. The results indicated that high levels of parent-reported SM were primarily associated with high levels of social anxiety symptoms. The number of spoken words was negatively related to behavioral inhibition: children with a more inhibited temperament used fewer words during the speech tasks. Future research is necessary to test whether the temperament characteristic of behavioral inhibition prompts children to speak less in novel social situations, and whether it is mainly social anxiety that turns this taciturnity into the psychopathology of SM.

## Introduction

Selective mutism (SM) is a psychiatric disorder typically occurring during childhood that is characterized by an absence of speech in specific public situations in which the child is expected to speak (e.g., school), while in other situations the child’s production of speech is apparently quite normal (e.g., at home). The latest edition of the diagnostic and statistical manual of mental disorders (DSM-5 [1]) specifies that the selective absence of speech should be present for at least 1 month. Further, the failure to speak should not be attributable to a lack of knowledge of, or discomfort with, the spoken language required in the social situation. Moreover, the disturbance is not better explained by a communication disorder (e.g., childhood-onset fluency disorder) and does not occur exclusively during the course of autism spectrum disorder, schizophrenia, or another psychotic disorder. Finally, there should be interference with daily functioning: the absence of speech hinders the child to function well at school or in social interactions.

Epidemiological studies indicate that SM is a relatively rare disorder: its prevalence rates vary between 0.03 and 1 % [2], depending on the population under investigation and the strictness of the diagnostic criteria that are employed. The mean age of onset of SM is usually before age 5 years, but the disturbance may not come to clinical attention until children enter school for the first time. Research in which children with SM are followed for a longer time period has shown that the disorder has a mean duration of 8 years, after which the key symptom (i.e., the absence of speech in specific situations) normally disappears [3]. This does not mean, however, that children no longer have problems. Studies have demonstrated that children who have previously suffered from SM, continue to have communication problems, perform less well at school or work, and display higher rates of psychiatric disorders later on in their development [3, 4].

In the past, this psychiatric condition was known as ‘voluntary aphasia’ [5] or ‘elective mutism’ [6], labels which both suggest that children with this condition intentionally choose not to speak in certain situations or with certain people. Later ‘elective’ was replaced by ‘selective’ [7, 8], which is more neutral about the child’s motives and puts the emphasis on the fact that the lack of speech only occurs in particular contexts or settings. In addition, a steadily increasing amount of research has made clear that anxiety—and social anxiety in particular—is a prominent feature of children with SM [9–11]. In keeping with this, DSM-5 [1] now lists SM among the anxiety disorders, underlining that children with this problem are wary of speaking rather than not wanting to speak in specific situations.

The fact that SM is currently considered as an anxiety disorder also opens new possibilities for research. Previously, when SM was still classified among ‘other disorders of infancy, childhood, and adolescence’ [7, 8] the condition was typically seen as a distinct diagnostic category. One implication of this point-of-view was that its scientific investigation was limited to children who suffered from this condition, which appeared quite difficult given the low prevalence of the disorder. For (childhood) anxiety a dimensional approach is common which assumes that the disorder represents the extreme end of a continuum ranging from mild and nonclinical deficits via subclinical problems to severe psychopathology [12, 13]. An important implication for research is that studies can also be conducted in the normal population as the non-clinical manifestation of the psychopathology has a similar appearance and is thought to be caused by the same underlying mechanisms as its clinical variant [14, 15].

Thus, the current study explored the phenomenon of SM in a non-clinical sample of children aged 3–6 years. SM levels were measured in two ways. First, children performed two speech tasks to assess their absolute amount of spoken words in a novel social situation. Second, parents completed a questionnaire to assess children’s frequency of failure to speak across various social settings. In addition, parents also filled in scales for measuring anxiety disorder symptoms and the temperament characteristic of behavioral inhibition in their offspring. The anxiety symptoms scale was included to examine the positive relationship between SM and anxiety, and especially social anxiety, which has been documented so frequently in clinical populations [9–11]. The behavioral inhibition measure was incorporated because this temperament characteristic is a well-known risk factor for the development of childhood anxiety disorders [16]. Moreover, as reticence in the presence of unfamiliar adults and lack of spontaneous speech with unknown persons are among the best indicators of behavioral inhibition [17], it seems plausible to assume that this temperament feature is also associated with SM [2, 9, 11]. Yet, surprisingly no study can be found in the literature to support this notion.

To recap, this study examined the relations between behavioral inhibition and (social) anxiety symptoms, on the one hand, and symptoms of SM, on the other hand, in a sample of young, non-clinical children. It was hypothesized that higher levels of behavioral inhibition and anxiety symptoms, and social anxiety symptoms in particular, would be accompanied by higher levels of SM. Further, besides a correlational analysis, we also carried out regression analyses to explore the unique relations of behavioral inhibition and (social) anxiety symptoms to SM symptomatology.

## Method

### Participants and Procedure

Participants were recruited via 21 elementary schools in Weert and Cranendonck, two municipalities in the South-Eastern part of the Netherlands, and Bree, a municipality in the Eastern part of Belgium. Information letters and consent forms were distributed among the parents of the 3- to 6-year-old children who were still in kindergarten. The parents of 63 children agreed to participate and returned the signed informed consent form to the researchers. Eventually, 57 children (20 boys and 37 girls; mean age = 4.98 years, *SD* = .74, range 3–6 years) and their parents (35 mothers, 2 fathers, and 10 both parents; mean age mothers = 36.26 years, *SD* = 4.38, range 26–57 years, and mean age fathers = 39.81 years, *SD* = 4.96, range 30–54 years) took part in the study. The vast majority of the children and parents were Caucasian (>95 %) and all of them were fluent in Dutch. Based on the educational levels of both parents the socioeconomic status of most families was estimated as medium (53 %) or high (40 %). None of the parents had divorced, so all families were still complete.

After receiving the signed consent forms, researchers contacted parents by email or telephone in order to make an appointment for the assessment session. In most cases, this session took place at the families’ home. In two cases this was not possible and so testing was done at a different location (i.e., school, experimenter’s home). The assessment was always conducted by two female experimenters (the second and third author) who closely followed a standardized protocol. In order to reduce the influence of external factors, children and parent(s) were always tested seated at a table in a quiet room, where no other people were present. First, the experimenters introduced themselves and gave a short overview of the assessment session. Then children engaged in the speech tasks. During the first part of these tasks parents assisted their children, but during the second part—when children were interviewed (see below)—parents were instructed not to interfere and to complete a set of questionnaires. When both the father and mother were present during the assessment, they completed the questionnaires together. The total assessment lasted for about 30 min, and the essential parts of the session (i.e., the speech tasks) were recorded by means of a Dictaphone.

### Assessment

The short version of the *Behavioral Inhibition Questionnaire* (BIQ-SF [18, 19]) is a 14-item parent-report instrument for assessing behavioral inhibition in preschool children. Item examples are “My child is shy when first meeting new children”, “My child gets upset when being left in new situations for the first time, for example kindergarten”, and “My child approaches new situations or activities very hesitantly”, which have to be answered on 6-point Likert scales, ranging from 1 (hardly ever) to 6 (almost always). A BIQ-SF total score is calculated by summing the scores on all items (range 14–84), with higher scores being indicative of higher levels of behavioral inhibition. The psychometric properties of the BIQ-SF are good [19, 20].

The *Preschool Anxiety Scale*-*Revised* (PAS-R [21]) is an adaptation of the Preschool Anxiety Scale (PAS [22]), a 30-item parent-based questionnaire for measuring symptoms of social anxiety, separation anxiety, generalized anxiety, specific fears, and obsessive–compulsive difficulties. Items (e.g., “My child worries that he/she will do something embarrassing in front of other people”, “My child would be upset at sleeping away from home”, “My child is frightened of dogs”) are rated on a 5-point scale ranging from 0 (not at all true) to 4 (very often true). As this study intended to make a comparison between social anxiety and non-social anxiety, we used the social anxiety subscale and a combined score of the remaining anxiety subscales. Adequate reliability and validity have been demonstrated for the PAS-R [21].

The *Selective Mutism Questionnaire* (SMQ [23]) is a 17-item parent-rating measure of children’s frequency of failure to speak across various settings including school and other public/social situations. Items such as “My child speaks in groups or in front of the class” and “My child speaks when in clubs, teams, or other organized activities outside of school” are scored on a 4-point scale with 0 = never, 1 = seldom, 2 = often, and 3 = always. A total score can be computed by summing scores on all items. Lower scores on the SMQ thus reflect low frequencies of speaking behavior, and thus higher symptom levels of SM. The SMQ is a reliable scale and its score has been shown to be predictive of SM diagnostic status [23, 24].

Children’s absolute amount of speech during a novel social situation was assessed by means of two *Speech Tasks*. For the first speech task, children were instructed to present a *monologue* about school. To give children some idea about what they were expected to do, (one of) their parent(s) provided an example by giving a brief talk about their favorite leisure time activity. Then, children were given a number of cues of what they could talk about during their monologue, such as the teacher, other children, activities inside the classroom and on the playground, after which they were invited to start their oral presentation. The second speech task was an *interview* consisting of eight open-ended questions (e.g., “What did you do during the summer holiday?” and “What did you do during your last birthday party?”) which were alternately posed by the two experimenters. For the first four questions no explicit instructions were given thereby giving an impression of children’s spontaneous way of responding to unknown persons. For the remaining four questions children were explicitly instructed to answer as elaborated as possible thereby providing an index of children’s maximal responding. Afterwards, both experimenters listened independently to the Dictaphone recordings to count the number of words spoken during the monologue and both parts of the interview.

### Data Analysis

The statistical package for social sciences (SPSS) was employed to perform the data analysis. First, we conducted *t-*tests to evaluate gender differences and computed correlations to study the influence of age. Second, to investigate the reliability of the study variables, internal consistency coefficients (Cronbach’s alphas) and (intraclass) correlations were computed. Third, the main research questions were examined by means of correlations and regression analyses. In the linear regression analyses, SM symptoms as measured by the SMQ and the total number of spoken words during the speech tasks were the dependent variables, while behavioral inhibition, social anxiety, and other anxiety symptoms were the predictors.

## Results

### General Findings

A number of significant gender differences were found. As can be seen in Table 1, parents reported that girls exhibited higher levels of behavioral inhibition [*t*(55) = 2.51, *p* < .05], social anxiety symptoms [*t*(52.41) = 2.72, *p* < .01], and SM [*t*(52.35) = 3.66, *p* < .01] than boys. (Marginally) significant correlations were found between children’s age, on the one hand, and SMQ scores (*r* = .25, *p* = .06) and total number of spoken words during the speech tasks (*r* = .25, *p* = .05), on the other hand. In other words, with increasing age, children used more words during the monologue and interview and tended to display lower levels of SM. Given these gender and age effects, we controlled for these demographic variables in the main analyses of this study. Further, the questionnaires that were completed by the parents (BIQ, PAS-R, and SMQ) showed good internal consistency: that is, all Cronbach’s alphas ranged between .81 and .91. The reliability of the speech tasks was also satisfactory: both experimenters counted an almost identical number of words (*r* = .99, *p* < .001), and the correlation between the numbers of words spoken during monologue and interview was so high (*r* = .68, *p* < .001) that we combined them into one total score.^1^

**Table 1:** Mean scores (standard deviations), gender differences, and reliability coefficients for measures that were used in this study

	Total group (*N* = 57)	Boys (*n* = 20)	Girls (*n* = 37)	Reliability^a^
BIQ behavioral inhibition	40.51 (11.83)	35.40 (8.71)	43.27 (12.46)*	.91
PAS-R social anxiety	10.82 (5.79)	8.40 (4.12)	12.14 (6.18)*	.88
PAS-R non-social anxiety	23.12 (10.10)	20.00 (8.47)	24.81 (10.61)	.81
SMQ selective mutism^b^	24.05 (6.81)	27.75 (4.68)	22.05 (7.00)*	.91
Speech tasks: number of spoken words	413.46 (772.78)	483.30 (299.57)	375.70 (253.39)	.68/.99

*BIQ* Behavioral Inhibition Questionnaire, *PAS*-*R* Preschool Anxiety Scale-Revised, *SMQ* Selective Mutism Questionnaire

* Significant gender difference at *p* < .05

^a^Reliability of questionnaires was assessed by means of Cronbach’s alpha. For the speech tasks, the correlation between the monologue and interview parts (left value) and the inter-rater correlation coefficient (right value) were computed

^b^Lower scores on the SMQ are indicative for higher symptom levels

### Correlations Among SM, Actual Amount of Speech, Behavioral Inhibition, and (Social) Anxiety

Partial correlations (corrected for gender and age) among all study variables are displayed in Table 2. Three conclusions can be drawn from this table. First, the temperament characteristic of behavioral inhibition was associated with higher symptom levels of social anxiety (*r* = .82), other anxiety disorders (*r* = .44), and SM (as indexed by the SMQ; *r* = −.64) as well as a lower number of spoken words during the speech tasks (*r* = −.56). Second, as hypothesized, social anxiety symptoms were more strongly associated with symptom levels of selective mutism and lower number of spoken words than non-social anxiety symptoms (*r*s being −.68 vs. −.27 and −.52 vs. −.27, respectively, *Z*s being 3.53, *p* < .001 and 1.94, *p* = .05, respectively). Third, a significant positive correlation was found between SMQ scores and number of spoken words during the speech tasks (*r* = .35): that is, children with higher SMQ scores, thus for whom parents indicated lower levels of SM symptoms, used more words during the monologue and interview.

**Table 2:** Partial correlations (corrected for gender and age) among various measures

	(1)	(2)	(3)	(4)
(1) BIQ behavioral inhibition				
(2) PAS-R social anxiety	.82***			
(3) PAS-R non-social anxiety	.44**	.42**		
(4) SMQ selective mutism^a^	−.64***	−.68***	−.27*	
(5) Observation: number of spoken words	−.56***	−.52***	−.27*	.35**

*N* = 57

*BIQ* Behavioral Inhibition Questionnaire, *PAS-R* Preschool Anxiety Scale-Revised, *SMQ* Selective Mutism Questionnaire

* *p* < .05; ** *p* < .01; *** *p* < .001

^a^Lower scores on the SMQ are indicative for higher symptom levels

### Unique Relations Between Behavioral Inhibition/(Social) Anxiety and SM

To examine unique relations between behavioral inhibition/(social) anxiety and SM, linear regression analyses (which controlled for gender and age on Step 0) were performed (see Table 3). The first analysis, in which SMQ scores were the dependent variable, showed that the three predictors together explained 38 % of the total variance [*F*(3,51) = 15.82, *p* < .001]. Note, however, that social anxiety was the only variable that made a unique significant contribution to parent-reported SM scores. The second analysis, in which number of spoken words during the speech tasks was the dependent variable, revealed that the three predictors together also accounted for a significant proportion of the variance (i.e., 29 %; *F*(3,51) = 8.03, *p* < .001). This time only the contribution of behavioral inhibition was marginally significant.

**Table 3:** Results of the linear regression analyses predicting SMQ scores (top panel) and number of spoken words during the speech tasks (bottom panel) from behavioral inhibition, social anxiety and non-social anxiety symptoms

	*B*	*SE*	*β*	*R* ^2^
SMQ selective mutism^a^				.38***
BIQ behavioral inhibition	−.15	.11	−.24	
PAS-R social anxiety	−.60	.22	−.45**	
PAS-R non-social anxiety	.02	.08	.02	
Number of spoken words				.29***
BIQ behavioral inhibition	−9.07	4.77	−.39*	
PAS-R social anxiety	−8.60	9.67	−.18	
PAS-R non-social anxiety	−.86	3.42	−.03	

*N* = 57

*BIQ* Behavioral Inhibition Questionnaire, *PAS*-*R* Preschool Anxiety Scale-Revised, *SMQ* Selective Mutism Questionnaire

* *p* = .06; ** *p* < .01; *** *p* < .001

^a^Lower scores on the SMQ are indicative for higher symptom levels. In both regression analyses, we controlled for age and gender on Step 0

## Discussion

The present study examined the relations between behavioral inhibition and (social) anxiety symptoms, and symptoms of SM in a sample of non-clinical children aged 3–6 years. The results of the correlational analyses indicated that behavioral inhibition was associated with higher symptom levels of social anxiety, other anxiety disorders, and SM, which is in agreement with a vast amount of literature showing that this temperament characteristic is a vulnerability factor for the development of anxiety pathology in children [16, 25]. More importantly, this is the first study providing straightforward empirical support for the relation between behavioral inhibition and SM. So far, research had only shown that children with SM display characteristics that seem to be indicative of an inhibited temperament, including shyness [26, 27], low sociability [28], withdrawal and low adaptability [29]. The fact that behavioral inhibition seems to be involved in SM points at a shared etiology with other childhood anxiety pathology, and thus supports the current DSM-5 classification of SM as an anxiety disorder [9].

Two additional points regarding the relationship between behavioral inhibition and SM can be made. First, the current data indicated that there was no direct relation between behavioral inhibition and SM. That is, regression analysis revealed that the contribution of this temperament factor to parent-reported SM symptoms was no longer significant when correcting for (social) anxiety symptoms. Maybe the link between behavioral inhibition and SM is more indirect and possibly mediated by (social) anxiety, which is a scenario that certainly warrants futher longitudinal investigation. Second, the point made above even makes more sense when acknowledging that there seems to be a logical inconsistency between behavioral inhibition as defined by wariness towards the unfamiliar and the impairment characteristic of SM which often involves familiar social partners (e.g., classmates) and situations (e.g., school, for a discussion, see [30, 31]).

The parent-rated index of SM correlated only moderately with the number of words spoken by children during the speech tasks. As already noted by Epstein [32, 33], it may be quite difficult to validate a psychological construct by means of an observational assessment. That is, observations performed on a single occasion provide only a limited sample of the child’s behavior, which may be strongly guided by momentary emotions and motives as well as situational characteristics. In contrast, when completing a questionnaire, the rater is able to take into account the child’s behavior in a variety of situations. Note in passing that the SMQ [23] indeed prompts parents to take into account various settings as items refer to the child’s speech behavior in school and other public/social situations. Meanwhile, parent ratings of behavioral inhibition and social anxiety symptoms did substantially correlate with the number of words used during the speech tasks implying that these characteristics were better indicators of children’s actual speech behavior than symptoms of SM. This suggests that the speech tasks were challenging enough to make children’s tendencies towards behavioral inhibition and social anxiety manifest. Full-blown symptoms of SM were probably relatively rare in this non-clinical sample and thus less clearly related to children’s actual amount of spoken words during the speech tasks. Moreover, it is possible that a liability to display symptoms of SM only becomes visible when the social situation is more stress-provoking.

Regression analysis demonstrated that behavioral inhibition was a better predictor of number of spoken words during the speech tasks than social anxiety (although its unique contribution was only borderline significant). This indicates that the correlation between social anxiety and actual amount of speech was mainly carried by the overlap between social anxiety and behavioral inhibition [34], and that it is primarily the temperament characteristic of behavioral inhibition that may prompt children to speak less in a novel social situation. On the basis of all these findings, one might also question the validity of the current speech tasks as an index of SM. Given the fact that this assessment was conducted at home—with both parents present, the tasks probably gave too little occasion for the child to exhibit symptoms of SM. In contrast, the tasks appeared to be sensitive enough to measure features of an inhibited temperament.

Interestingly, another regression analysis with SMQ scores as the dependent variable revealed that social anxiety—and not behavioral inhibition or other anxiety symptoms—was the only significant, independent predictor of parent-reported SM symptoms. Assuming that the SMQ is an index for measuring the more psychopathological manifestations of SM, this finding confirms previous studies showing that there exists an intimate relation between social anxiety and SM. For example, several studies have found that almost all children with SM also fulfill the diagnostic criteria of social anxiety disorder [35, 36]. Some authors have even argued that the condition should be seen as an extreme symptom of this anxiety disorder [35], while others have proposed that SM is an early childhood variant of social phobia [37]. Good arguments can be advanced for both positions. For instance, studies showing that children with SM exhibit even higher levels of social anxiety symptoms than children with social phobia [38–40] of course support the extreme symptom hypothesis, whereas the early age-of-onset of SM in combination with the fact that complete muteness normally tends to disappear with increasing age [3, 4] is in favor of the young child variant hypothesis (for a discussion, see [41]).

Two additional findings of the present study deserve some comment. First, significant gender differences were found for a number of variables: more specifically, girls displayed higher levels of behavioral inhibition, social anxiety, and SM symptoms as compared to boys. Although this result is in keeping with the common notion that girls are more anxiety-prone and display higher levels of anxiety problems [42] as well as SM [36] than boys, it is also true that at a preschool age such gender differences are not always documented [20, 22]. Second, small but significant relations were found between age, on the one hand, and SMQ scores and number of spoken words during the speech tasks, on the other hand. This indicates that SM symptomatology decreased and the amount of spoken words during the monologue and interview tasks increased as children were older. With the progression of age and in its wake cognitive and language development it is obvious that children are better capable of verbally expressing themselves, and this may also at least in part account for the clinical observation that SM tends to dissipate over time [3, 4].

It should be acknowledged that the current investigation suffers from a number of limitations. First of all, 24 elementary schools were involved in this study, with the parents of more than 850 children being invited to participate, implying that the response rate of this study was quite low (i.e., <7 %). Further, by accident the sample also contained almost twice as many girls than boys, which of course raises further questions concerning the representativeness of the sample. Second, it is important to keep in mind that the design of the present study was correlational in nature. Although there were theoretical reasons for considering behavioral inhibition and (social) anxiety as predictors of SM symptoms (as tested in the regression analyses), one should be cautious with drawing conclusions in terms of cause-effect relations among these variables. Third, only parents completed rating scales for measuring children’s level of behavioral inhibition, (social) anxiety, and SM symptoms, and so the possibility cannot be ruled out that the obtained pattern of results was influenced by shared method variance. Preferably, research on child psychopathology should adopt a multi-informant perspective, and for example, in this study teachers could have provided important cross-validational information by completing a similar set of questionnaires. Fourth, a number of other factors of interest were not considered in this study or the analyses; these include: children’s language development and frequency of social contacts, and parents’ verbal skills, communication with the child, rearing behaviors, or psychopathology [e.g., (social) anxiety]. Fifth, in relation to the previous shortcoming, this study only explored SM in relation to anxiety pathology. In the literature, SM has also been associated with other types of child psychopathology such as communication disorders and externalizing problems [2, 9], and so it would have been interesting if we had included non-anxiety measures in this study. Finally, the speech tasks only consisted of two parts (i.e., monologue and interview), which were conducted at home in the presence of the parent(s) by two friendly experimenters. As noted earlier, the testing situation may be been insufficiently provoking for the typical symptoms of SM to emerge. Future studies should systematically explore the influence of situational characteristics (e.g., familiar vs. unfamiliar place, parents present vs. absent, experimenter behaving in a friendly vs. ambiguous way) on children’s amount of speech behavior, anxiety levels, and SM symptomatology.

## Summary

In spite of these shortcomings, the present study clearly demonstrates that there are clear relationships between behavioral inhibition/social anxiety and SM symptoms, which is in agreement with notions that have been formulated in the extant literature [2, 9]. The findings are in keeping with the notion that anxiety seems to be a prominent feature or correlate of SM, although they remain silent with regard to the debate whether SM should be regarded as a separate anxiety disorder or merely represents a prodromal or extreme version of social anxiety disorder [41]. The latest edition of the DSM [1] has clearly chosen for the former option, but obviously the latter possibilities need further scientific exploration. Meanwhile, the results are encouraging as they seem to indicate that the dimensional approach of psychopathology also applies to SM, which makes it possible to study the condition in non-clinical populations. Longitudinal research would be particularly welcome to explore the precise role of an inhibited temperament and social anxiety in the development of young children who remain partially or even fully silent in specific social situations.

## Footnotes

1 Number of spoken words during the spontaneous responding and maximal responding parts of the interview correlated .86 (*p* < .001).
